# Supplementary material for: Spatiotemporal variations of “triple-demic” outbreaks of respiratory infections in the United States in the post-COVID-19 era
Source: BMC Public Health. 2023 Dec 7;23:2452. doi: 10.1186/s12889-023-17406-9 (PMC10704638; doi:10.1186/s12889-023-17406-9)
Supplement: Supplementary file 1 — Supplementary Material 1 [file 12889_2023_17406_MOESM1_ESM.docx]

**Spatiotemporal Variations of “Triple-demic" Outbreaks of Respiratory Infections in the United States in the Post-COVID-19 Era**

**Spatiotemporal patterns of space time clusters in February 2023**

**Table A1. Space-time univariate clusters of COVID-19, influenza, and RSV from October 2022 to February 2023 (RR = relative risk).**

| **Cluster** | **Duration** | **P value** | **Diseases** | **Observed** | **Expected** | **RR** | **Total state (N)** | **States (RR>1)** |
| --- | --- | --- | --- | --- | --- | --- | --- | --- |
| 1 | 2023.01.30-2023.02.27 | <.001 | COVID-19 | 5170 | 104695.02 | 0.049 | 1 | 0 |
| 2 | 2022.12.19-2023.02.27 | <.001 | COVID-19 | 1344668 | 1011763.56 | 1.41 | 14 | 12 |
| 3 | 2023.01.23-2023.02.27 | <.001 | COVID-19 | 310983 | 483025.72 | 0.63 | 16 | 3 |
| 4 | 2022.12.26-2023.02.27 | <.001 | COVID-19 | 230912 | 148356.35 | 1.58 | 4 | 3 |
| 1 | 2023.01.02-2023.02.27 | <.001 | influenza | 448116 | 4935213.88 | 0.081 | 13 | 3 |
| 2 | 2023.01.09-2023.02.27 | <.001 | influenza | 404088 | 4545098.70 | 0.080 | 18 | 11 |
| 1 | 2022.12.19-2023.02.27 | <.001 | RSV | 3213 | 27901.60 | 0.10 | 15 | 1 |
| 2 | 2023.01.09-2023.02.27 | <.001 | RSV | 5937 | 20735.85 | 0.26 | 10 | 3 |

**Table A2. Space-time multivariate clusters from October 2022 to February 2023 (RR = relative risk).**

| **Cluster** | **Duration** | **P value** | **Type** | **Diseases** | **Observed** | **Expected** | **RR** | **Total state (N)** | **States (RR>1)** |
| --- | --- | --- | --- | --- | --- | --- | --- | --- | --- |
| 1 | 2023.01.02-2023.02.27 | <.001 | Twindemic | RSV | 2463 | 22900.50 | 0.096 | 13 | 1 |
|  |  |  |  | influenza | 448116 | 4935213.88 | 0.081 |  | 3 |
| 2 | 2023.01.09-2023.02.27 | <.001 | Triple-demic | RSV | 10957 | 21090.28 | 0.49 | 18 | 10 |
|  |  |  |  | influenza | 404088 | 4545098.70 | 0.080 |  | 11 |
|  |  |  |  | COVID-19 | 563631 | 745205.75 | 0.73 |  | 5 |


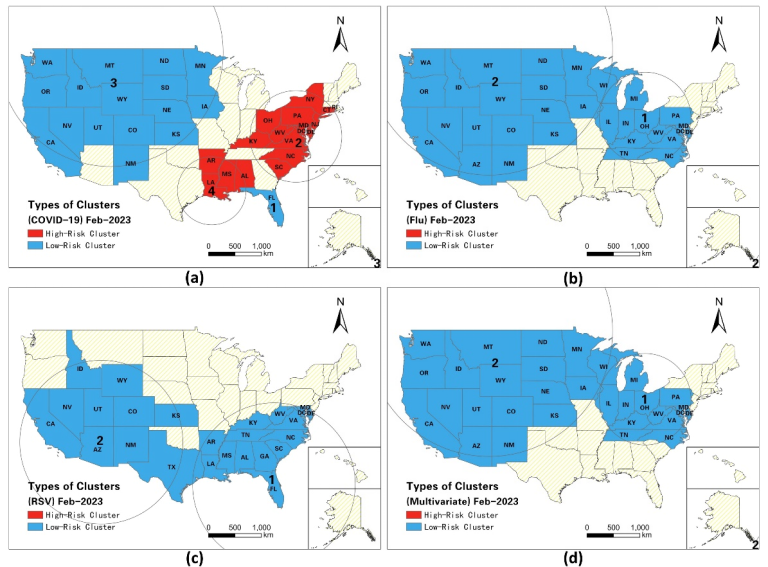


**Figure A1. Spatial Patterns of space-time clusters in February 2023.**

**Spatiotemporal Propagation of Univariate Clusters**

**Table A3. Space-time univariate clusters from October 2022 to January 2023 (RR = relative risk).**

| **October 2022** | | | | | | | | |
| --- | --- | --- | --- | --- | --- | --- | --- | --- |
| **Cluster** | **Duration** | **P value** | **Diseases** | **Observed cases** | **Expected cases** | **RR** | **Total state (N)** | **States (RR>1)** |
| 1 | 2022.10.24-2022.10.31 | <.001 | COVID-19 | 73010 | 158543.20 | 0.43 | 11 | 2 |
| 2 | 2022.10.24-2022.10.31 | <.001 |  | 119601 | 70330.50 | 1.77 | 8 | 8 |
| 3 | 2022.10.31-2022.10.31 | <.001 |  | 89235 | 64616.01 | 1.41 | 10 | 8 |
| 4 | 2022.10.24-2022.10.31 | <.001 |  | 82971 | 108621.41 | 0.75 | 8 | 1 |
| 5 | 2022.10.24-2022.10.31 | <.001 |  | 2433 | 4615.73 | 0.53 | 2 | 0 |
| 1 | 2022.10.24-2022.10.31 | <.001 | Influenza | 2411008 | 500030.70 | 7.28 | 8 | 7 |
| 2 | 2022.10.31-2022.10.31 | <.001 |  | 748172 | 269284.44 | 3.02 | 11 | 6 |
| 3 | 2022.10.24-2022.10.31 | <.001 |  | 370929 | 616738.07 | 0.58 | 15 | 1 |
| 4 | 2022.10.24-2022.10.31 | <.001 |  | 24677 | 118063.99 | 0.21 | 2 | 0 |
| 5 | 2022.10.24-2022.10.31 | <.001 |  | 24743 | 111877.21 | 0.22 | 6 | 0 |
| 1 | 2022.10.24-2022.10.31 | <.001 | RSV | 6158 | 1174.19 | 5.80 | 6 | 5 |
| 2 | 2022.10.24-2022.10.31 | <.001 |  | 2990 | 749.09 | 4.17 | 1 | 1 |
| 3 | 2022.10.24-2022.10.31 | <.001 |  | 863 | 91.25 | 9.60 | 1 | 1 |
| 4 | 2022.10.24-2022.10.31 | <.001 |  | 2717 | 5268.89 | 0.49 | 10 | 3 |
| 5 | 2022.10.24-2022.10.31 | <.001 |  | 1912 | 774.33 | 2.52 | 2 | 2 |
| 6 | 2022.10.24-2022.10.31 | <.001 |  | 487 | 134.55 | 3.64 | 1 | 1 |
| **October 2022 - November 2022** | | | | | | | | |
| **Cluster** | **Duration** | **P value** |  | **Observed** | **Expected** | **RR** | **Total state (N)** | **States (RR>1)** |
| 1 | 2022.11.07-2022.11.28 | <.001 | COVID-19 | 6663 | 71080.24 | 0.09 | 1 | 0 |
| 2 | 2022.11.07-2022.11.28 | <.001 |  | 115342 | 62614.82 | 1.88 | 1 | 1 |
| 3 | 2022.11.21-2022.11.28 | <.001 |  | 141764 | 93142.01 | 1.55 | 5 | 3 |
| 4 | 2022.11.07-2022.11.28 | <.001 |  | 54274 | 96580.11 | 0.55 | 1 | 0 |
| 5 | 2022.11.07-2022.11.28 | <.001 |  | 74911 | 108128.23 | 0.68 | 6 | 1 |
| 6 | 2022.11.07-2022.11.28 | <.001 |  | 259899 | 222591.77 | 1.19 | 8 | 7 |
| 1 | 2022.11.07-2022.11.28 | <.001 | Influenza | 6583436 | 3064396.37 | 2.56 | 15 | 7 |
| 2 | 2022.11.21-2022.11.28 | <.001 |  | 3813598 | 1607061.06 | 2.62 | 14 | 8 |
| 1 | 2022.11.07-2022.11.28 | <.001 | RSV | 44983 | 17172.05 | 3.47 | 16 | 10 |
| 2 | 2022.11.07-2022.11.28 | <.001 |  | 4231 | 14495.37 | 0.27 | 10 | 1 |
| 3 | 2022.11.07-2022.11.28 | <.001 |  | 6113 | 2060.83 | 3.06 | 1 | 1 |
| 4 | 2022.11.07-2022.11.28 | <.001 |  | 3188 | 2043.33 | 1.57 | 2 | 2 |
| **October 2022 - December 2022** | | | | | | | | |
| **Cluster** | **Duration** | **P value** |  | **Observed** | **Expected** | **RR** | **Total state (N)** | **States (RR>1)** |
| 1 | 2022.11.21-2022.12.26 | <.001 | COVID-19 | 13853 | 123720.31 | 0.11 | 1 | 0 |
| 2 | 2022.12.05-2022.12.26 | <.001 |  | 526409 | 332588.83 | 1.67 | 10 | 8 |
| 3 | 2022.11.21-2022.12.26 | <.001 |  | 501971 | 324240.83 | 1.63 | 5 | 4 |
| 4 | 2022.12.26-2022.12.26 | <.001 |  | 25438 | 4281.95 | 5.97 | 1 | 0 |
| 1 | 2022.11.21-2022.12.26 | <.001 | Influenza | 9256761 | 5476524.48 | 1.90 | 19 | 10 |
| 2 | 2022.11.21-2022.12.26 | <.001 |  | 6138816 | 3135314.54 | 2.13 | 9 | 3 |
| 3 | 2022.12.12-2022.12.26 | <.001 |  | 537311 | 1344600.13 | 0.39 | 8 | 1 |
| 1 | 2022.11.21-2022.12.26 | <.001 | RSV | 12443 | 1427.95 | 9.31 | 1 | 1 |
| 2 | 2022.11.21-2022.12.26 | <.001 |  | 3838 | 20019.55 | 0.17 | 10 | 0 |
| 3 | 2022.11.21-2022.12.26 | <.001 |  | 6907 | 1030.34 | 6.94 | 1 | 1 |
| 4 | 2022.11.21-2022.12.26 | <.001 |  | 7326 | 2543.82 | 2.96 | 3 | 3 |
| 5 | 2022.11.21-2022.12.26 | <.001 |  | 648 | 4680.20 | 0.14 | 2 | 0 |
| 6 | 2022.11.21-2022.12.26 | <.001 |  | 414 | 2416.27 | 0.17 | 2 | 0 |
| **October 2022 - January 2023** | | | | | | | | |
| **Cluster** | **Duration** | **P value** |  | **Observed** | **Expected** | **RR** | **Total state (N)** | **States (RR>1)** |
| 1 | 2022.12.05-2023.01.30 | <.001 | COVID-19 | 1293259 | 870811.86 | 1.62 | 14 | 10 |
| 2 | 2022.12.05-2023.01.30 | <.001 |  | 484071 | 350418.17 | 1.41 | 1 | 1 |
| 3 | 2023.01.02-2023.01.30 | <.001 |  | 170974 | 265773.40 | 0.63 | 16 | 2 |
| 4 | 2022.12.26-2023.01.30 | <.001 |  | 155047 | 93638.16 | 1.67 | 4 | 2 |
| 5 | 2023.01.30-2023.01.30 | <.001 |  | 6609 | 29928.87 | 0.22 | 1 | 0 |
| 1 | 2023.01.02-2023.01.30 | <.001 | Influenza | 366760 | 3316453.98 | 0.10 | 13 | 3 |
| 2 | 2023.01.09-2023.01.30 | <.001 |  | 297871 | 2748867.62 | 0.10 | 18 | 11 |
| 1 | 2022.12.05-2023.01.30 | <.001 | RSV | 4768 | 27185.10 | 0.15 | 12 | 1 |
| 2 | 2022.12.05-2023.01.30 | <.001 |  | 9170 | 1701.19 | 5.61 | 1 | 1 |
| 3 | 2022.12.05-2023.01.30 | <.001 |  | 5602 | 1227.50 | 4.67 | 1 | 1 |
| 4 | 2023.01.02-2023.01.30 | <.001 |  | 5928 | 15433.12 | 0.36 | 8 | 3 |
